# Neuroprotective role of rice bran extract and its constituents in a neuroinflammatory mouse model

**DOI:** 10.1186/s12906-025-05097-1

**Published:** 2025-10-02

**Authors:** Sarah M. Abou El-Nagah, Mohammad Abdel-Halim, Ola A. Heikal, Reham M. AbdelKader

**Affiliations:** 1 Department of Pharmacology and Toxicology, Faculty of Pharmacy and Biotechnology, German University in Cairo, Cairo, 11835 Egypt; 2 Department of Pharmaceutical Chemistry, Faculty of Pharmacy and Biotechnology, German University in Cairo, Cairo, 11835 Egypt; 3 Narcotic, Ergogenic and Toxins Department, National Research Center, Cairo, Egypt

**Keywords:** Peroxisome-proliferator-activated-receptor-gamma (PPARγ), Alzheimer’s disease, CD36, EPA, DHA

## Abstract

**Background:**

Peroxisome proliferator-activated receptor gamma (PPARγ) is a nuclear receptor known to play a critical role in regulating neuroinflammation and neurodegenerative processes, including Alzheimer’s disease. Prior studies from our group demonstrated that rice bran extract (RBE) enhances cognitive function and increases PPARγ DNA-binding activity in the brain, effects that were abolished by PPARγ antagonism. These findings suggest that bioactive constituents within RBE may modulate PPARγ signaling. The current study aimed to provide additional evidence for the involvement of PPARγ activation in the neuroprotective effects of RBE and to identify key RBE-derived components that may contribute to these effects.

**Methods:**

A neuroinflammatory mouse model was treated orally for 21 consecutive days with RBE. The brain CD36 and amyloid-beta (Aβ) protein levels were measured. HPLC and GC were used to assess the levels of RBE components. To measure alterations in fatty acid content after treatment with RBE, brain levels of DHA, EPA and AA were assessed using UHPLC/MS-MS.

**Results:**

RBE treatment increased the brain levels of CD36, the direct PPARγ target, and decreased Aβ levels. A strong correlation was detected between the Aβ and CD36 protein levels. As RBE was found to be rich in linolenic acid (ALA), linoleic acid (LA) and oleic acid, their metabolites concentrations in mice brain were measured, and results indicated higher concentration of EPA and DHA after RBE treatment.

**Conclusions:**

RBE exerts neuroprotective effects potentially through activation of the PPARγ pathway, as evidenced by CD36 upregulation and Aβ reduction. The enrichment of RBE in polyunsaturated fatty acids (PUFAs), along with the observed increase in their brain-penetrant metabolites (EPA and DHA), suggests these lipids may contribute to the cognitive benefits of RBE.

**Supplementary Information:**

The online version contains supplementary material available at 10.1186/s12906-025-05097-1.

## Background

Neuroinflammation plays a key role in many neurodegenerative disorders, such as Parkinson’s disease and Alzheimer’s disease (AD) [1]. The nuclear receptor peroxisome proliferator-activated receptor gamma (PPARγ) acts as a key player in inflammation [2], and recent evidence has demonstrated that activation of PPARγ receptors attenuates the inflammatory response [3, 4]. Accordingly, thiazolidinediones (TZDs) (FDA-approved oral antidiabetic medications), which are known PPARγ agonists, have been evaluated in several clinical trials in AD patients. TZDs were found to alleviate brain inflammation symptoms and exert neuroprotective effects [5]. A study by Cheng et al. showed that pioglitazone treatment resulted in enhanced cognition and a reduction in amyloid-beta (Aβ) and tau pathology in AD patients [6]. Later, a phase III study in 2018 was terminated because it failed to meet its primary objective [7]. Furthermore, long-term TZD treatment has severe side effects, including edema, weight gain and cardiac hypertrophy [8–12]. Although TZDs have not continued their development in AD, they have shed light on a new target, the PPARγ pathway. Currently, a new compound, T3D-959, which is not a TZD but a PPARγ agonist, is undergoing clinical trials, and the first trials showed potential improvements in cognition [9]. A phase 2 trial evaluating its safety and efficacy in mild-moderate AD ended in February 2023, and the results are still unpublished [13].

PPARγ activation is associated with decreased inflammatory responses, prevention of neural cell death and suppression of microglial and astrocytic activation [4, 14, 15]. It was also reported to regulate numerous genes involved in the inflammatory process and the scavenger receptor CD36, which is a fatty acid transporter that transports FAs across the blood‒brain barrier [16]. CD36 has also been reported to be expressed on microglia and macrophages and causes phagocytosis of Aβ [17] Therefore, targeting PPARγ seems to be a promising tool.

Many natural products have been previously used and tested for their ability to improve cognition and overcome neuroinflammation [18–20]. Among these, polyunsaturated fatty acids (PUFAs) are well-recognized natural ligands of PPARγ and have been associated to anti-inflammatory and neuroprotective effects [21]. Rice bran extract (RBE), an alcoholic extract obtained during rice milling, is notably rich in PUFAs—including linoleic, linolenic, and oleic acids—in addition to a range of bioactive phytochemicals such as γ-oryzanol, tocopherols, and tocotrienols, all of which have been independently reported to influence PPARγ activity [22–24]. Our previous studies demonstrated that standardized RBE enhances memory and cognition, and modulates neuroinflammatory responses, effects that were significantly diminished in the presence of the PPARγ antagonist GW9662. These findings support the hypothesis that RBE exerts its neuroprotective effects, at least in part, through direct engagement with the PPARγ pathway [25–28]. Importantly, the PUFAs in RBE are recognized as selective PPARγ modulatory activators (SPPARMs)—partial agonists that mimic endogenous ligands and are associated with a reduced risk of side effects compared to full agonists like thiazolidinediones [21, 29]. This favorable profile positions RBE as a promising, naturally derived, and potentially safer alternative for PPARγ modulation.

In the current study we provide further evidence for the possible involvement of PPAR-γ activation in the neuroprotective effect of RBE and investigate the accumulation of RBE constituents in brains of treated mice in an attempt to identify the components contributing to the cognitive enhancement of RBE. The first aim of this study was to investigate the effect of RBE on CD36, a direct target of PPARγ. Moreover, since CD36 causes phagocytosis of the Aβ protein [30], we examined the effect of RBE on Aβ levels in the brains of RBE-treated mice. Finally, since PUFAs are major components of RBE extracts and are reportedly PPARγ agonists, it is plausible that they might contribute to this effect, especially that CD36, the fatty acid translocase is involved in transporting fatty acids across the blood-brain barrier.

## Materials and methods

### Chemicals

The RBE used was provided by Health Tec Company (Healthtech^®^) through project collaboration partially funded by the Academy of Scientific Research and Technology (ASRT – Egypt). γ-Oryzanol, α-tocopherol, γ-tocopherol, α-tocotrienol, and γ-tocotrienol were purchased from Sigma Aldrich (Germany) and were dissolved in ethanol as 100 mM stock solutions. LPS (strain 055: B5) was purchased from Sigma‒Aldrich, Germany. Arachidonic acid (C20:4 *n* = 6; AA), docosahexaenoic acid (C22: 6 *n* = 3; DHA), and eicosapentaenoic acid (C20:5 *n* = 3; EPA) standards and D8-AA (C20:4 − 6 d8) were purchased from Cayman Chemical, USA. Mouse Amyloid Beta Peptide 1–42 (Aβ1–42) ELISA Kit and Mouse CD36 ELISA Kit were purchased from My BioSource, USA.

### Preparation of stabilized RBE

The extract was prepared by subjecting rice bran to high temperature for a short time (HTST) to inactivate the lipase enzyme, followed by maceration with 1:3 w/v 95% alcohol at 50 °C overnight for three successive extraction sessions to obtain the ethanolic extract. Then, the filtrate was filtered and concentrated under vacuum to produce the RBE, which was stored in the refrigerator to be warmed in a water bath at 37 °C with sonication just before use.

### Phytochemical profile of RBE

The bioactive compound of RBE, γ-oryzanol, was determined as reported previously [26]. The vitamin E profile of RBE was quantified using HPLC as previously reported with minor modifications. Briefly, Dionex-Ultimate 3000–Thermo Fisher HPLC was used, and separation was performed using a reversed-phase column (Phenomenex Kinetex PFP Column 2.6 μm 150 × 4.6 mm) provided with a C-18 guard column maintained at 40 °C. The elution was linear isocratic using methanol: H_2_O (85:15) as the mobile phase at a flow rate of 0.8 ml/min and a diode array as the detector. Peaks were recorded and integrated using Thermo Scientific Chromeleone software.

### Fatty acid profile in RBE

The RBE fatty acid profile was analyzed using GC-FID (Agilent 7890B), and separation was performed using a Zebron ZB-FAME column (60 m × 0.25 mm internal diameter × 0.25 *µ*m film thickness). Hydrogen was used as the carrier gas at a flow rate of 1.8 ml/min in split-1:50 mode, and the temperature program was 100 °C for 3 min, increased at 2.5 °C/min to 240 °C and held for 10 min. The injector and detector (FID) were held at 250 °C and 285 °C, respectively.

### Animals and treatment

Adult male Swiss albino mice weighing 20 to 30 g were used. Mice were purchased from the National Centre of Research (NRC-Cairo, Egypt), housed in the German University in Cairo (GUC) animal house, and allowed to settle and adapt for 1 week before injection procedures. Constant temperature and humidity conditions were maintained, as were 12 h light/dark cycles. The animals had free access to food and water. The LPS model was developed according to Lee et al.., where a daily I.P. injection of LPS dissolved in saline at a dose of 250 µg/kg body weight was injected for seven consecutive days [31–33]. This mouse model was previously characterized and showed a decrease in cognition, increase in brain Aβ levels and inflammatory mediators [26, 31, 34, 35]. Briefly, the mice were divided into four groups. Each group consisted of 7 mice, and this number was based on our previous experience with similar studies: control (I), RBE (II) LPS (III), and RBE + LPS (IV). The control group received vehicle only for 21 consecutive days. The groups receiving RBE (II, IV) were orally administered RBE (100 mg/kg dissolved in 0.6% DMSO) by oral gavage daily for 21 consecutive days. The mice in the groups injected with LPS (III, IV) were injected I.P daily during the last week only (15th day–21st day). All mice were weighed daily throughout the course of the study. The used dose and duration of treatment was chosen based on our previous studies, using this design and showing efficacy and no toxicity [25, 26, 28]. After the injection procedure, the mice were sacrificed on the 21 st day by cervical dislocation. To avoid interference with the study outcomes, the use of anesthetics was avoided since they were reported to affect memory and cognitive functions [36]. On the other hand, cervical dislocation is a physical method for rapid sacrifice and is completely safe for the brain [37, 38]. The brains were excised, the cerebellum was removed, and the brain was cut into two hemispheres; each hemisphere was washed with saline and stored in a 2 ml polypropylene tube at −80 °C until further use. One hemisphere was used for the determination of Aβ and CD36 levels, and the other hemisphere was used for the determination of PUFAs. Values lower than the standard detection limits were excluded from the study.

All animal procedures were approved by the Ethics Committee of the German University in Cairo (Ethical approval number PTX-2017-03-RA) and were performed in accordance with the National Institutes of Health (NIH) Guide for the Care and Use of Laboratory Animals and with ARRIVE guidelines.

### Determination of CD3protein levels using ELISA

A mouse CD36 ELISA kit was used to determine the concentration of CD36 protein in the experimental groups. The procedure was performed according to the manufacturer’s protocol. The plate was placed in a multilabel counter, and the optical density (OD) was measured at 450 nm.

### Determination of Aβ_1-42_

Aβ was quantified using a mouse Aβ_1−42_ ELISA kit. The procedure was performed according to the manufacturer’s protocol. The sample’s OD was measured at 450 nm in a V-630 microplate reader.

### Determination of PUFA levels in mouse brains

Concentrations of DHA, AA, and EPA in the brains of treated and untreated mice were obtained as described by [39]. Calibration curves were generated for standards of AA, DHA, and EPA using AA-d_8_ as an internal standard.

#### Sample preparation: lipid extraction

Brain tissue** (**100–150 mg) was weighed on ice to prevent further fatty acid metabolism and then quickly transferred to a 10 ml glass tissue homogenizer. Citric acid buffer followed by methanol and chloroform (1:1:3) were added, and the brains were homogenized on ice. The homogenate was then transferred to glass tubes, vortexed for 15 s, and centrifuged at 4 °C at 2200 × g for 6 min. It is vital that the procedure is performed very quickly and on ice to prevent the formation of additional branched fatty acid esters of hydroxy fatty acids. Then, the chloroform layer was transferred to another glass tube. Finally, the samples were subjected to evaporation in a concentrator for 50 to 60 min and then stored at −80 °C until analysis. The brain samples were resuspended in 140 µL of methanol and centrifuged for 5 min before injection into the UHPLC‒MS/MS system.

#### UHPLC-MS/MS experimental conditions

The UHPLC‒MS/MS method for determination of the fatty acids of interest was performed using arachidonic acid-d8 (AA-d8) as an internal standard. Analyses were performed on a Waters ACQUITY Xevo TQD system, which consisted of an ACQUITY UPLC H-Class system and a Xevo TQD triple-quadrupole tandem mass spectrometer with an electrospray ionization (ESI) interface (Waters Corp., Milford, MA, USA). A CORTICS C18 50 mm × 4.6 mm column (particle size, 2.7 μm) was used to separate the analytes (Waters, Wexford, Ireland). A gradient elution at a flow rate of 0.3 mL/min was conducted for chromatographic separation using 5 mM ammonium acetate in water (A) and acetonitrile (B). The gradient was run as follows:

**Table:** 

Time (min)	% A	% B
0.00	30	70
3.00	10	90
3.01	5	95
4.00	0	100
5.00	30	70
7.00	30	70

The dwell time was automatically set by MassLynx 4.1 software. The column temperature was set at 40 °C. Nitrogen was used as the desolvation and cone gas at flow rates of 1000 and 10 L/h, respectively. Argon was used as the collision gas at a pressure of approximately 3.67 × 10 − 3 mbar. The optimal MS parameters were as follows: capillary voltage, 2.85 kV; radio frequency (RF) lens voltage, 2.5 V; source temperature, 150 °C; and desolvation gas temperature, 500 °C. The ESI source was operated in negative mode. Quantification was performed using cone voltage, multiple reaction monitoring (MRM) transitions and collision energy presented in Table 1.

**Table 1:** MS parameters for developed method

Compound name	Parent (m/z)	Daughter (m/z)	Cone voltage (V)	Collision energy (V)
AA	303.10	259.10	50	12
DHA	327.17	283.25	42	10
EPA	301.19	257.19	44	12
AA-d8	311.29	267.36	50	12

#### Preparation of PUFA calibration curves

For the calibration curve, a set of serial dilutions of each standard was prepared. For DHA, the concentrations used were 0.2, 0.4, 0.8, 1, 2, 4, 8, and 10 µg/ml; for AA, the concentrations used were 0.2, 0.4, 0.8, 1, 2, 4, and 8 µg/ml. For EPA, the concentrations prepared were 0.01, 0.02, 0.04, 0.08, 0.1, 0.2, 0.4, 0.8, and 1.6 µg/ml. The calibration curve points were prepared by adding 50 µl of each fatty acid dilution to 50 µl of the brain matrix obtained from the control mouse (prepared as in the sample preparation section), and 5 µl of 80 µg/ml AA-d_8_ (IS) was added. The calibration curve samples were run using the developed UHPLC-MS/MS method described above. For each fatty acid (AA, DHA, and EPA), the area ratios (Y) were plotted against the respective fatty acid concentration, and a linear calibration curve was obtained. For sample measurements, the concentrations of fatty acids were derived from their respective constructed calibration curves (supplementary figures S4 and S5).

### Statistical analysis

Statistical analysis was performed using the Instant automated software GraphPad Prism 9. The results are expressed as the means ± SEMs. The results were analyzed using one-way analysis of variance (ANOVA) followed by Sidak’s multiple comparisons test. *P* ≤ 0.05 indicated a statistically significant difference. Graphical representation of the results was generated using Graph Pad Prism 9 software, and significant changes are shown on the graph. For correlation analysis, Pearson correlation was used. The sample size for this study was determined using free G*Power software. The effect size based on previous similar studies should be set between 0.6 and 0.8. A desired statistical power of 0.80 and a significance level of 0.05 were selected. These parameters resulted in an estimated sample size of 7 animals per group.

## Results

### The effect of RBE on CD36 and Aβ1–42 protein levels

**Fig. 1:**
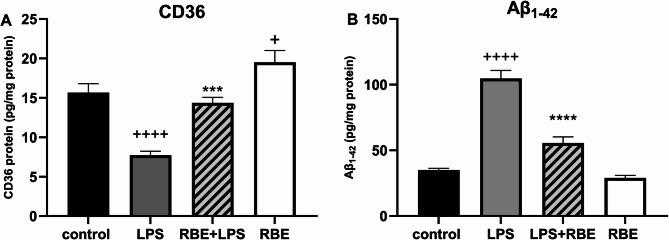
Effect of RBE treatment on CD36 and Aβ_1-42_ in mouse brains. Mice were pretreated orally for 21 consecutive days with RBE, while LPS was injected in the last week only. Oral RBE treatment increased CD36 protein levels (Fig. 1A) and decreased _Aβ1‒42_ protein levels (Fig. 1B) in the neuroinflammatory model. LPS administration decreased CD36 levels compared to those in the control group, and pretreatment with RBE significantly reversed this effect (Fig. 1A). However, LPS administration increased Aβ_1‒42_ protein levels, and this effect was reversed by RBE pretreatment. The data are presented as the means ± SEMs. *n* = 6–7/group. One-way ANOVA was performed followed by Sidak’s multiple comparison test. +*p* ≤ 0.05, ++++*p* ≤ 0.0001 compared to the control, ****p ≤ 0.0001 and ***p ≤ 0.001compared to LPS. No significant differences were observed between the RBE and control groups

To determine whether PPARγ is affected by RBE treatment, as previously suggested by our group, the levels of the direct target CD36 were measured in the brains of the treated mice. Pretreatment with rice bran extract (RBE) (100 mg/kg/day for 21 days) in a neuroinflammatory LPS mouse model (055:250 µg/kg/day B5 for 7 days) resulted in a significant increase in CD36 levels compared to those in the LPS-injected group. Moreover, the administration of RBE alone significantly increased CD36 protein levels, while LPS injection resulted in a significant decrease in CD36 levels compared to those in the control group (Fig. 1A).

As CD36 has a direct effect on Aβ_1−42_, we examined the effect of RBE on Aβ _1−42_ deposition. LPS administration significantly increased Aβ_1−42_ protein concentration in the mice brains. However, the administration of RBE prior to LPS significantly protected against these effects compared to those in the LPS group. Finally, RBE administration alone had no effect on the Aβ _1−42_ level (Fig. 1B).

### Phytochemical and fatty acid profile of RBE

Our next step was to examine the levels of active constituents in our RBE and the levels of PUFAs, which may contribute to the effect on PPARγ. The levels of γ-oryzanol (mg/g) and vitamin E congeners (µg/g) in RBE were quantified using HPLC and derived from the calibration curves of their corresponding standards (supplementary figures S1 and S2). The γ-oryzanol content in the RBE sample was 25.7 mg/g (2.5%). α-Tocopherol, γ-Tocopherol, α-Tocotrienol, and γ-Tocotrienol content were comparable to previously reported data on RBE [40, 41] α-Tocopherol, 97.5; γ-Tocopherol, 180; α-Tocotrienol, 34; γ-Tocotrienol; 267.5(µg/g) (supplementary Table S1). The percentages of fatty acid esters present in the RBE were estimated and are shown in Table 2. The contents of unsaturated fatty acids were comparable to those previously reported (supplementary figure S3).

**Table 2:** Percentages of fatty acid esters present in the RBE

Peak	RT	Name	Area	Area Sum %
1	18.803	Myristic acid	65451.75	0.3
2	24.684	Palmitic acid	3,800,328	17.13
3	25.73	Palmitoleic acid	33480.55	0.15
4	30.277	Stearic acid	398888.4	1.8
5	31.077	Oleic acid	8,981,630	40.5
6	32.712	Linoleic acid	8,287,774	37.37
7	34.703	Linolenic acid	261973.5	1.18
8	35.536	Arachidic acid	169565.8	0.76
9	36.139	*cis*−11-Eicosenoic acid	121428.5	0.55
10	40.525	Behenic acid	58853.14	0.27

### PUFA levels in the brains of mice

**Fig. 2:**
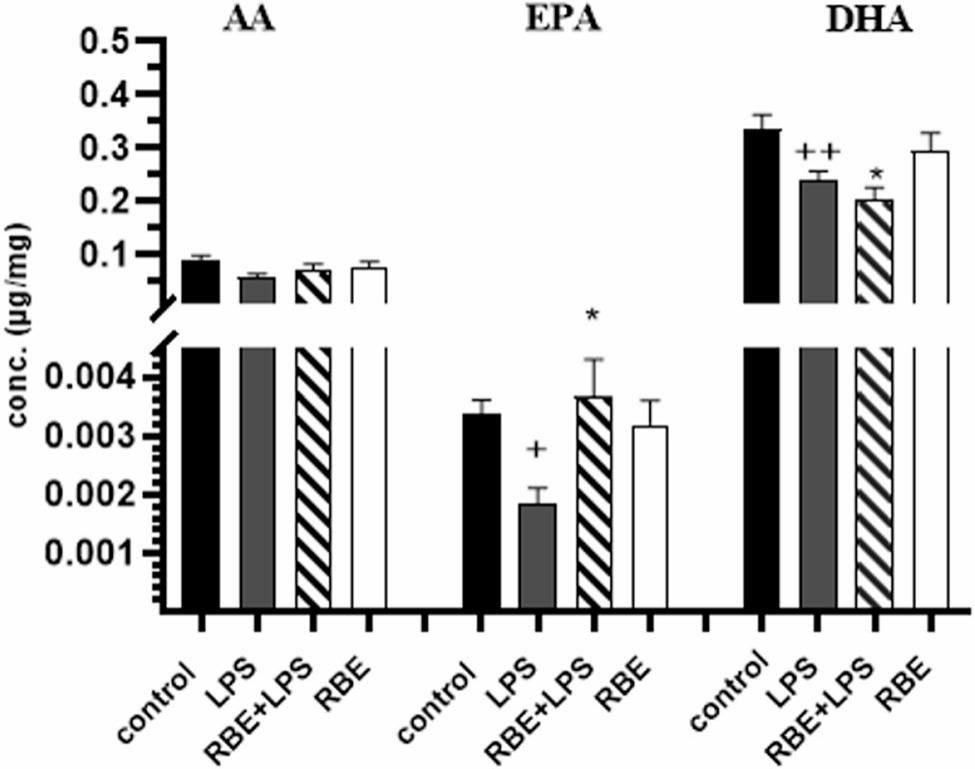
Concentrations of DHA, EPA, and AA in the brains of treated mice. Mice were pretreated orally for 21 consecutive days with RBE, while LPS was injected in the last week only. LPS decreased the DHA and EPA levels, while RBE pretreatment significantly reversed this effect. RBE showed no effect on AA concentration in LPS mouse model. RBE administration to normal mice had no effect on DHA, EPA or AA. One-way ANOVA was performed followed by Sidak’s multiple comparison test. Mean ± SEM. *N* = 6–7/group. +*p* ≤ 0.05, ++*p* ≤ 0.01 compared to control, *p ≤ 0.05, compared to LPS

Finally, we wanted to test whether RBE treatment can alter the levels of PUFAs in the brain. The results showed a significant decrease in the DHA and EPA levels in the LPS-treated mice compared to those in the control mice. Interestingly, this effect was significantly reversed in the LPS group pretreated with RBE compared to the LPS group (Fig. 2A and B). AA levels were not affected in our mouse model or by RBE treatment (Fig. 2C) (Supplementary Figure S4).

### Correlations between CD36, Aβ, and brain fatty acids

Since CD36 is known to act as a direct scavenger of Aβ, as well as a fatty acid translocator, we aimed to test whether the levels of CD36 in the mouse brain can be correlated with Aβ levels and/or the measured levels of fatty acids. Interestingly, a highly significant negative correlation was detected between CD36 and Aβ, but no significant correlation was detected between CD36 and any of the measured brain fatty acids. These results are summarized in Table 3.

**Table 3:** Correlations between CD36 levels and Aβ and between CD36 levels and brain fatty acid levels

CD36 correlations	Aβ_1-42_	DHA	EPA	AA
r	−0.793	0.165	0.263	−0.193
r2	0.629	0.027	0.069	0.037
P value	1.361e-006****	0.463	0.249	0.378

Cd36 brain levels showed a highly significant negative correlation with Aβ_1−42_ in the brains of mice. DHA, EPA and AA did not correlate with CD36 levels.****p ≤ 0.0001

## Discussion

Neuroinflammation has been strongly implicated in neurodegenerative disorders. Recently, we studied the effect of RBE on cognitive function in a neuroinflammatory mouse model. Our previous studies reported that RBE possesses significant anti-inflammatory and neuroprotective effects, as well as the ability to enhance mitochondrial function, primarily through modulation of the PPAR-γ pathway [25–27]. The use of the PPAR-γ antagonist GW9662 markedly reduced the anti-inflammatory actions of both RBE and pioglitazone (a known PPAR-γ agonist), supporting the hypothesis that RBE acts via direct PPAR-γ activation. Furthermore, when comparing RBE to synthetic PPARγ agonists such as pioglitazone (a thiazolidinedione), our previous studies showed that RBE exerted comparable anti-inflammatory and cognitive-enhancing effects. Both RBE and pioglitazone shifted microglial polarization toward an anti-inflammatory phenotype and upregulated key markers such as CD36 [28]. Importantly, while pioglitazone was associated with significant weight gain—a common TZD side effect—RBE-treated groups maintained stable body weights, suggesting a more favorable metabolic safety profile [26].

Moreover, since RBE is rich in polyunsaturated fatty acids (PUFAs), which are recognized as promising partial agonists of PPAR-γ receptors, this further supports the possible classification of RBE components as partial PPAR-γ modulators, similar to endogenous ligands like fatty acid and eicosanoids [22, 29]. Thus, RBE may provide a naturally derived, safer alternative to synthetic PPARγ modulators.

In the present study, we examined the mechanism by which RBE affects the PPARγ signaling pathway by investigating the levels of CD36, which is directly modulated by the PPARγ receptor. Interestingly, RBE treatment increased the CD36 protein level in normal mice and reversed the LPS-induced CD36 decrease in our mouse model. Since an increase in CD36 levels is linked to an increase in Aβ phagocytosis [42–45], we concurrently examined the levels of Aβ_1‒42_. As expected, the decrease we observed in CD36 protein levels in the LPS-injected group was concurrent with an increase in Aβ levels in the same group, and this decrease was reversed in the RBE-pretreated group and was concurrent with a decrease in Aβ_1−42_. To the best of our knowledge, this is the first study to report an increase in CD36 protein levels induced by RBE in both normal and neuroinflammatory mouse brains (Fig. 1). However, these results are in accordance with our previously reported findings by El-Din et al.., who reported that RBE was able to increase the mRNA levels of CD36 in a neuroinflammatory model and decrease Aβ_1−42_ [28]. Although no other studies have reported the effect of RBE on the CD36 protein, few studies have indicated an effect of some of the components of RBE on CD36. For instance, Dong et al. reported an increase in cortical and hippocampal protein expression of CD36 following fish oil and vitamin E supplementation in APP/PS1 mice [46]. Moreover, active components of RBE, such as γ-oryzanol, have also been reported to reduce the protein levels of Aβ- in the cerebral region in an STZ-induced AD mouse model [47]. In addition, DHA, a metabolite of ALA present in RBE, was also reported to decrease Aβ production, aggregation, and clearance in individuals with moderate dementia and AD [48].

These results support our hypothesis that RBE components acts on PPARγ. Others have reported that in mice overexpressing the amyloid precursor protein, treatment with rosiglitazone, a high-affinity agonist of PPARγ, upregulated CD36 and facilitated Aβ clearance [49].

The observed increase in CD36 in our study, which acts as a fatty acid translocator [50], might enhance the uptake and transport of PUFAs in the brain. Furthermore, PUFAs activate PPARγ, which can lead to a detected increase in CD36 levels. Given that RBE is rich in PUFAs, the next step was to measure the levels of PUFAs and their metabolites in the brains of the RBE-treated mice in an attempt to correlate the findings of CD36 with the levels of the PUFAs. A complete fatty acid profile was performed for the RBE extract to determine the most abundant PUFAs in our extract. Our findings revealed linolenic acid (ALA), linoleic acid (LA), and oleic acid (omega-9 FA) with estimated concentrations of 1.18%, 37.37%, and 40.5%, respectively, in the RBE total fatty acid content. Since our results indicate that RBE is rich in PUFAs and that it affects the levels of the fatty acid translocator CD36, which allows fatty acids to cross the BBB, it was plausible to measure the levels of brain fatty acids after treatment with RBE. Accordingly, the concentrations of DHA; docosahexaenoic acid (C22: 6 *n* = 3), EPA, eicosapentaenoic acid (C20:5n = 3) and AA; Arachidonic acid (C20:4 *n* = 6) (metabolites of ALA, LA)that reach the brain were measured. DHA and AA were chosen because they constitute the major concentrations of ω−3 (ALA) and ω−6 (LA) in the brain, respectively [50] and they were found to be altered in the brains of AD patients [51]. Encouraging reports in the field have associated ω−3 FA intake with memory improvement. This was reported in healthy adults who showed improved memory function after DHA supplementation, as well as in reducing the risk of developing AD or cognitive decline [52, 53]. Furthermore, both EPA and DHA have been reported to be potent inducers of CD36 mRNA levels [54].

Compared with those of the control group, the brain samples of the LPS group showed significant decrease in the concentrations of DHA and EPA, while the AA concentration did not change compared with that of the control group (Fig. 2). Interestingly, in the groups treated with RBE prior to LPS, the brain concentrations of EPA and DHA were greater than those in the LPS group, while the AA concentration did not change, suggesting that RBE plays a role in reversing the LPS effect by restoring EPA and DHA concentrations in brain tissues. This finding is in agreement with the reported effect of DHA in alleviating the deleterious effects of LPS [55, 56]. Indeed, previous studies have indicated alterations in PUFA concentrations after treatment with rice bran oil in plasma or other tissues [57–59], but to the best of our knowledge, brain PUFA concentrations after RBE administration have not been previously studied.

Since previous reports have indicated that EPA and DHA alter CD36 levels [54] and stimulate the phagocytosis of Aβ42 [60], our results indicate that the effects observed on CD36 and Aβ42 after RBE treatment could be attributed to the increase in brain EPA and DHA levels caused by RBE. Additionally, emerging evidence suggests that EPA and DHA are more potent activators of PPARγ compared to LA, making the observed increases in brain EPA and DHA concentrations after RBE treatment particularly relevant to the neuroprotective mechanisms we propose [21, 22].

Finally, it is worth mentioning that the administration of RBE alone to healthy mice did not alter brain EPA, DHA or AA levels, indicating alterations in their levels only in the neuroinflammatory model.

Our current findings add to the growing body of evidence that RBE-derived PUFAs and their metabolites, which reach the brain, contribute to the modulation of neuroinflammatory conditions. A previous study examining the neuroprotective effects of RBE reported that α-tocopherol was the most abundant vitamin E derivative detected in guinea pig brains following RBE administration, while α- and γ-tocotrienols were present only in trace amounts [61]. In NMRI aged mice treated with RBE, no significant differences in brain α-tocopherol levels were observed between young and aged groups, and other vitamin E derivatives were below detection limits [27, 62]. These findings suggest that α-tocopherol and other vitamin E congeners are unlikely to solely account for the neuroprotective effects associated with RBE in aging. Moreover, studies investigating RBE’s effects on microglial activation demonstrated that, although RBE treatment modulated cytokine production (PGE2, TNF-α, IL-1, IL-6) in activated microglia, α-tocopherol alone did not significantly alter cytokine levels, indicating that RBE’s effects on neuroinflammation extend beyond the activity of α-tocopherol alone [41]. Collectively, despite differences in experimental models and endpoints, these studies highlight the likely synergistic action of multiple bioactive constituents within RBE. and highlighted the impact of our study in further identifying other RBE constituents which could be responsible for the beneficial neuroprotective effects.

In conclusion, we previously reported that RBE increases cognitive function and that this effect is attenuated by a PPARγ antagonist, indicating a possible role for the PPARγ receptor. In this study, we confirmed that CD36, a direct target of PPARγ, was modulated by RBE, which led to a decrease in Aβ levels. Furthermore, we showed that PUFAs are a major component of RBE and that treatment with RBE alters brain concentrations of EPA and DHA. Since PUFAs are known PPARγ modulators, at least part of the effect of RBE may be attributed to them. Further studies are required to understand the exact role of PUFAs in the cognitive enhancement effect of RBE. This is a current limitation to the current study, and will be addressed in upcoming research.

Our future research will specifically investigate the potential interaction of RBE-derived PUFAs with the nuclear factor kappa B (NF-κB) signaling pathway, a known downstream target of PPARγ modulation. Recent evidence highlights the PPARγ/AMPK/SIRT1 axis as a key regulatory pathway in inflammation. Given that PUFAs have been shown to activate AMPK, it is plausible that RBE may exert its anti-inflammatory and neuroprotective effects via the PPARγ/AMPK/SIRT1/NF-κB axis. This hypothesis will be the focus of upcoming studies to define the specific molecular pathways involved in the cognitive and anti-inflammatory effects of RBE.

## Supplementary Information

Supplementary Material 1

## Data Availability

All required data is provided within the manuscript and supplementary files.
