# Supplementary material for: Neuroprotective role of rice bran extract and its constituents in a neuroinflammatory mouse model
Source: BMC Complement Med Ther. 2025 Oct 2;25:351. doi: 10.1186/s12906-025-05097-1 (PMC12490112; doi:10.1186/s12906-025-05097-1)
Supplement: Supplementary file 1 — Supplementary Material 1 [file 12906_2025_5097_MOESM1_ESM.docx]

**Supplementary Data**

**Role of Rice Bran extract and its constituents in neuroprotection in a neuroinflammatory mouse model**

*Sarah M. Abou El-Nagah^1^, Mohammad Abdel-Halim^2^, Ola A. Heikal^3^, Reham M. AbdelKader^1^*

^1^Department of Pharmacology, Toxicology and Clinical Pharmacy German University

^2^Department of Pharmaceutical Chemistry, Faculty of Pharmacy and Biotechnology, German University in Cairo, Cairo 11835, Egypt.
^3^Narcotic, Ergogenic and Toxins department, National Research Center


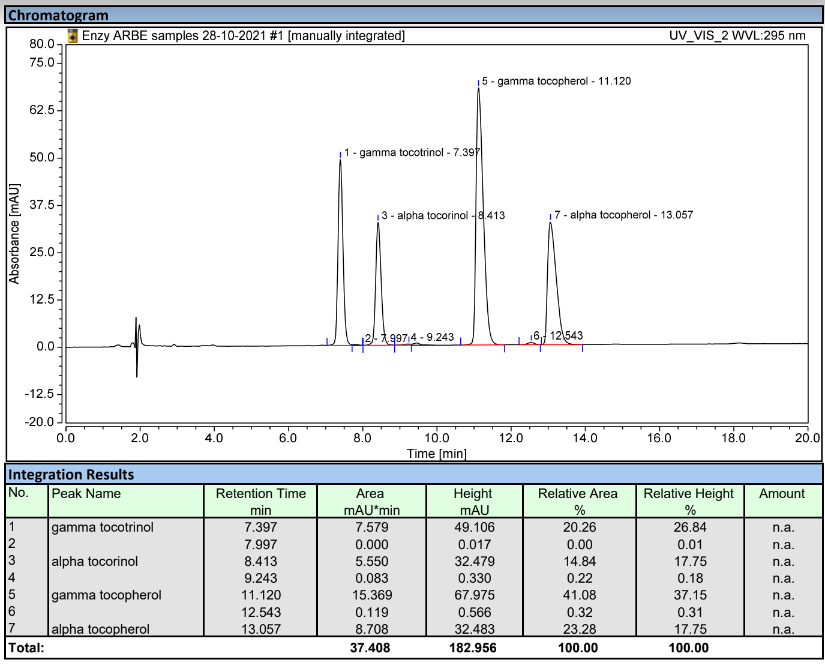


Figure S1: HPLC chromatogram of total Vitamin E congener’s standards

**Figure S2: Calibration curves of γ vitamin E isomers. γ and α Tocopherols and Tocotrienols**

Table S1: Concentrations of γ-Oryzanol (mg/g) and Vitamin E congeners (µg/g) in RBE A.

| ***Rice Bran extract*** | **γ – Tocopherol**  **(µg/g)** | **α -Tocopherol**  **(µg/g)** | **γ – Tocotrienol**  **(µg/g)** | **α -Tocotrienols**  **(µg/g)** | **γ-Oryzanol (mg/g)** |
| --- | --- | --- | --- | --- | --- |
| ***RBE*** | \|  \| \| --- \|   180.4 | 97.4 | 267.5 | 34.8 | 25.7 |


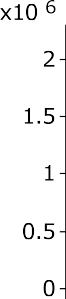

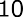

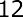

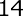

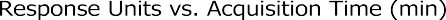

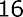

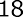

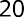

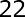

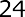

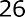

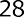

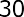

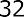

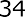

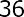

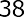

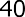

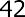

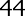

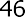

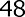

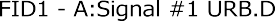


**Figure S3: GC chromatogram of Fatty acids profile**

Figure S4: MRM chromatograms of the measured fatty acids and the internal standard

(A) Deuterated arachidonic acid (AA,C20:4n6, (B) Arachidonic acid (AA) C20:4n6, (C) Decosahexanoic acid (DHA) C22:6n3, (D) Eicosapentanoic acid (EPA) C20:5n3.

Figure S5: Standard calibration curves of EPA, DHA and AA obtained by UHLPC-MS/MS
